# Management of Dysphagia in Nursing Homes: A National Survey

**DOI:** 10.1007/s00455-021-10275-7

**Published:** 2021-03-04

**Authors:** Mina C. N. Engh, Renée Speyer

**Affiliations:** 1 Department Special Needs Education, University of Oslo, Oslo, Norway; 2 Indre Østfold Municipality, Askim, Norway; 3 School of Occupational Therapy, Social Work and Speech Pathology, Faculty of Health Sciences, Curtin University, Perth, Australia; 4 Department of Otorhinolaryngology and Head and Neck Surgery, Leiden University Medical Centre, Leiden, The Netherlands

**Keywords:** Assisted living facility, Questionnaire, Eating and drinking, Deglutition disorder, Swallowing problems, Norway

## Abstract

The management of dysphagia may differ by country and clinical setting. The purpose of this study was to describe the management and care pathways for elderly people with dysphagia in nursing homes across Norway using an online survey. A national survey was developed that consisted of 23 questions covering various areas related to dysphagia care in nursing homes: background information of respondents, nursing homes, and residents and staff; screening and assessment of dysphagia including use of specialist consultation; management, practice patterns, and interventions targeting residents with dysphagia; training of staff; and perceived quality of current clinical practices in their nursing home. A total of 121 respondents completed the online survey, resulting in an overall response rate of 23.2%. Substantial discrepancies in dysphagia management were identified between nursing homes. In approximately 75% of nursing homes, residents were not routinely screened or assessed for swallowing problems. Although nursing homes used a broad range of strategies and routines for people with eating and swallowing difficulties, bolus modification seemed standard practice. Oral hygiene strategies were lacking in over 80% of nursing homes, and almost 50% did not have access to external experts, including speech therapists. Although nursing home staff rated the overall quality of care for people with eating and swallowing problems as high, their rating seemed mainly based on care for malnutrition and not directly aimed at dysphagia. The survey identified an evident need for training and upskilling staff in Norwegian nursing homes and raising awareness of the serious consequences and comorbidities that can result from dysphagia.

**Supplementary Information:**

The online version contains supplementary material available at 10.1007/s00455-021-10275-7.

## Introduction

Oropharyngeal dysphagia (OD) presents a serious health threat. It may result in failure to maintain hydration and nutrition and pose a risk of choking and aspiration, with devastating consequences [1, 2]. Dysphagia also affects quality of life and is associated with social and psychological burden, anxiety, and depression [3–5].

Oropharyngeal dysphagia affects people all over the world, with prevalence data in the general population that vary between 2.3 and 16% [6], depending on definitions of swallowing problems and methods used for screening and assessment [6–8]. The condition is especially common in elderly, stroke patients, and in those with neurological diseases [4] and is considered a major health care problem in nursing home residents [2]. The high prevalence of dysphagia among the elderly in an aging population also has a major impact on health economics and the health care system [9]. OD is considered a geriatric syndrome, as it is highly prevalent among older people, is caused by multiple factors, is associated with several comorbidities and poor prognosis, and needs a multidimensional approach to be treated [9].

Although dysphagia in the elderly population has a high incidence and the consequences are severe, dysphagia is often under-diagnosed in vulnerable or hospitalized patient populations [4, 10]. Additionally, diet modifications and restrictions on oral intake in residents in nursing homes are frequently prescribed without further assessment of underlying causes of suspected swallowing problems [8, 11]. Nursing homes provide care to many vulnerable populations frequently suffering from multiple comorbidities [7]. A cross-sectional survey study repeated over several years among residents in nursing homes in nineteen countries from Europe and North America reported dysphagia prevalence data of 13.4% [12]. However, when using clinical screening to evaluate the presence of dysphagia in older adults in nursing homes, the prevalence was estimated to be as high as 52.7% [2]. A systematic review [8] reported dysphagia prevalence data in long-term care ranging from 7 to 40%, while the percentage of long-term residents who were malnourished ranged from 12 to 54%.

Clinical symptoms of OD are associated with an increased risk of malnutrition in nursing home residents [7]. One-year national registry data in Norway confirmed that 50.5% of all nursing homes screened residents were at nutritional risk [13]. Of all residents screened, 36.1% were at risk for malnutrition; however, data on dysphagia have been lacking. To date, no studies have provided data on the prevalence, screening and assessment, or management of and interventions for dysphagia in nursing homes in Norway.

The purpose of this study is to describe the management and care pathways for elderly people with dysphagia in nursing homes across Norway using a national survey. Our study focused on background information of respondents, nursing homes, and residents and staff; screening and assessment for eating and swallowing difficulties (dysphagia), including use of specialist consultation; management of interventions for residents with eating and swallowing difficulties; and training of the nursing home employees and their perceived quality of current clinical practices in their nursing home.

## Methods

### Survey Development

An online survey using Nettskjema was developed based on the current literature and input from experts (including clinicians, managers at nursing homes and academics with expertise in instrument development). Nettskjema is a tool for designing and conducting online surveys specifically designed to meet Norwegian privacy requirements. The tool was developed and is operated by the University Information Technology Center (USIT) at the University of Oslo, Norway. The survey was piloted among four content experts, after which minor modifications were made based on their feedback to improve the uniform interpretability of the survey questions.

The final survey consisted of 23 questions covering various areas related to dysphagia care in nursing homes (see Supplementary information): background information of respondents, nursing homes, and residents and staff (8 items); screening and assessment of dysphagia including use of specialist consultation (67 items); management, practice patterns, and interventions targeting residents with dysphagia (54 items); training of staff (3 items); and perceived quality of current clinical practices in their nursing home (1 item). The survey contained short explanations where appropriate on topics such as ‘dysphagia’ or ‘screening and assessment’. As participants were expected to have different educational backgrounds and possibly be less familiar with medical terms, we aimed to avoid the use of professional jargon. As such, after having briefly introduced the concept of dysphagia, the term ‘dysphagia’ was replaced with ‘eating and swallowing difficulties’ in the actual survey. Participants could elaborate on questions using open comment boxes throughout the survey. The questionnaire consisted of multiple choice questions (17 items), matrix questions (4 items), a numeric textbox question (1 item), and one ordinal scale (1 item).

### Recruitment of Participants

The IPLOS register (Individbasert Pleie- og Omsorgs Statistikk) from Statistics Norway (Statistisk sentralbyrå) was consulted in January 2018 to retrieve an overview of nursing homes per county in Norway; 807 businesses were registered as nursing homes. Psychiatric nursing homes were not included in the study.

All 807 nursing homes were considered eligible for participation in our survey. During the first contact by phone, a standardized informative introduction was used with brief information about the survey’s purpose and content. The nursing homes that could not be contacted with the first call were called once more. One staff member per nursing home was invited to participate. Invited staff members should be engaged in health care through clinical and/or managerial tasks or responsibilities and have knowledge about management and daily routines within their nursing home.

Next, upon registering contact e-mail addresses from nursing homes willing to participate in our survey, additional information including a link to the online survey was sent. The participants received an information letter with the purpose of the survey, ethical considerations and information about privacy and informant rights. The survey was open for respondents between November 2019 and February 2020, during which time, potential participants received up to three reminders.

### Data Analysis

Raw data were exported from Nettskjema into Excel. After data cleaning and quality assessment, the data were imported into SPSS (version 26, Chicago, IL). For multiple choice options, the responses supplied by the participants in open text boxes were coded to an existing response option if applicable or coded as a new response option. Descriptive analyses were performed to provide frequency and percentage distributions. Chi-square tests were used to investigate differences between groups of respondents. To reduce the initial number of variables in the multivariate regression analyses, a series of univariate chi-square tests was used as the pre-selection process [14]. Only variables with chi-square *p*-values of less than 0.1 were considered for inclusion in backward elimination binary logistic regression analyses to identify variables that best contributed to the regression model [14].

## Results

### Participants

Out of the 807 nursing homes contacted, 536 (66.4%) homes agreed to participate. 15 e-mail deliveries failed, resulting in 521 valid contacts. A total of 121 participants completed the online survey, resulting in an overall response rate of 23.2% (121/521). Table 1 presents the number of invited nursing homes versus the total number of nursing homes per county in Norway and a response rate based on the number of invited nursing homes per county. The response rate per county ranged between 6.9 (Oslo) and 36.0% (Vestland).

**Table 1:** Nursing homes per county in Norway versus response rate

County in Norway (alphabetical order)	Number of invited^a^ versus total number of nursing homes per county (%)	Number of survey respondents	Response rate^b^ (%)
Agder (Vest-Agder og Aust-Agder)	32/53 (60.4%)	7	21.9
Innlandet (Hedmark og Oppland)	29/65 (44.6%)	8	27.6
Møre og Romsdal	49/61 (80.3%)	14	28.6
Nordland	39/61 (63.9%)	10	25.6
Oslo	29/43 (67.4%)	2	6.9
Rogaland	47/68 (69.1%)	12	25.5
Troms og Finnmark	53/64 (82.8%)	9	17.0
Trøndelag	60/86 (69.8%)	12	20
Vestfold og Telemark	35/58 (60.3%)	10	28.6
Vestland (Hordaland og Sogn og Fjordane)	75/109 (68.8%)	27	36.1
Viken (Østfold, Akershus og Buskerud)	73/139 (52.5%)	10	13.7
**Totals**	**521/807 (64.6%)**	**121**	**23.2**

Total numbers appear in bold.

^a^E-mail invitation upon agreement per phone.

^b^Ratio of respondents versus invitees.

Background information on the participants is provided in Table 2. Most of the respondents were clinical nurses and department managers (57.0%). Other respondents were chief executive officers (13.2%), directors of care (14.9%), and heads of allied health (9.9%). More than half of all respondents (55.4%) had a health care professional background. The other respondents were trained in health management (22.3%) or were specialized health care professionals (21.5%).

**Table 2:** Position and professional background of respondents

Position	Number of participants per professional background (%)				Total number of respondents (%)
	Health management	Health care professionals	Specialized health care professionals	Other background	
Chief Executive Officer	5	10	1	0	**16 (13.2%)**
Director of Care	6	10	2	0	**18 (14.9%)**
Clinical Nurse/Dept. Manager	16	38	14	1	**69 (57.0%)**
Head Allied Health	0	5	7	0	**12 (9.9%)**
Other staff	0	4	2	0	**6 (5.0%)**
Total number of respondents (%)	**27 (22.3%)**	**67 (55.4%)**	**26 (21.5%)**	**1 (0.8%)**	**121 (100%)**

Total numbers appear in bold.

### Nursing Homes and Staffing

**Fig. 1:**
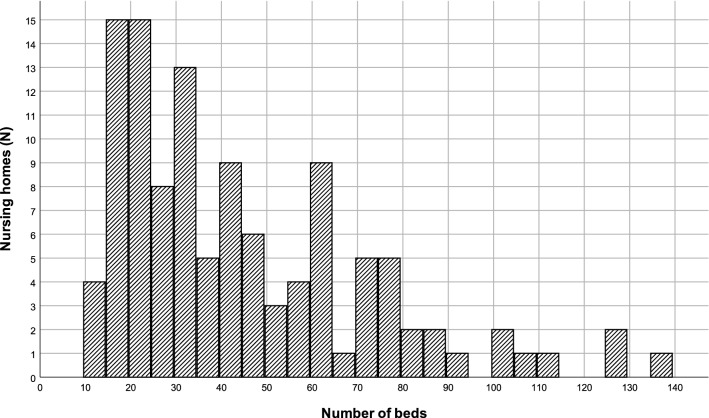
Number of beds per nursing home

Nursing homes offered different types of care, including daycare, short-term stay and long-term stay. Almost all homes offered long-term stays (97.5%); 10.7% (13/121), and 34.7% (42/121) of nursing homes offered daycare and short-term stays, respectively (> 5 places). Six homes had limited numbers of beds for other care, such as observation or respite care. The participating nursing homes showed great variation in the number of beds (Fig. 1), ranging between 12 and 135 beds with a median of 35 beds (interquartile range: 23–60). The most frequent diagnostic groups (> 5 residents) were dementia (94.2%; 114/121) and post-stroke (42.2%; 51/121). Only very few nursing homes housed people with traumatic brain injury, head and neck or oesophageal cancer, or congenital neurological conditions. Homes offering care for people with dementia (n = 114) showed the following distribution in the numbers of residents diagnosed with dementia: 50.0% (57/114) ranging between 6 and 20 residents, 29.0% (33/114) ranging between 21 and 30 residents, and 21.0% (24/114) with more than 50 residents; 88.2% (45/51) of homes caring for post-stroke residents (*n* = 51) estimated the numbers of post-stroke residents between 6 and 20, whereas 11.8% (6/51) estimated numbers between 21 and a maximum of 40 residents.

Table 3 provides numbers and types of staff per nursing home (full-time equivalent), including managerial positions, health care professionals, and unskilled personnel. All nursing homes had nurses and care assistants among their staff, whereas other health care professionals were less frequently appointed in homes: occupational therapists, 53.2%; physiotherapists, 37.4%; speech therapists, 7.7%; social workers, 6.5%; and nutritionists, 6.3%.

**Table 3:** Staffing in nursing homes: staff numbers per position for all nursing homes (frequency table)

Staff	Number of staff (FTE)											Number of respondents
	0	1–4	5–9	10–19	20–29	10–39	40–49	50–59	60–69	70–79	≥ 80	
Management	3	94	15	0	0	0	0	0	0	0	0	112/121 (92.6%)
Nurse/Social educator	0	5	31	42	29	6	5	0	0	0	0	118/121 (97.5%)
Care assistant^a^	0	1	14	44	19	17	8	5	4	0	4	116/121 (95.9%)
Social worker	72	4	1	0	0	0	0	0	0	0	0	77/121 (63.6%)
Physiotherapist	57	33	1	0	0	0	0	0	0	0	0	91/121 (75.2%)
Occupational therapist	44	47	3	0	0	0	0	0	0	0	0	94/121 (77.7%)
Speech therapist	72	6	0	0	0	0	0	0	0	0	0	78/121 (64.5%)
Nutritionist	74	5	0	0	0	0	0	0	0	0	0	79/121 (34.7%)
Kitchen staff	18	56	22	4	0	0	0	0	0	0	0	100/121 (82.6%)
Unskilled personnel^b^	12	19	18	14	13	7	4	1	1	2	3	94/121 (77.7%)

*FTE* Full time equivalent

^a^Healthcare workers and social workers

^b^Care worker and unskilled assistant

### Screening and Assessment for Dysphagia

**Fig. 2:**
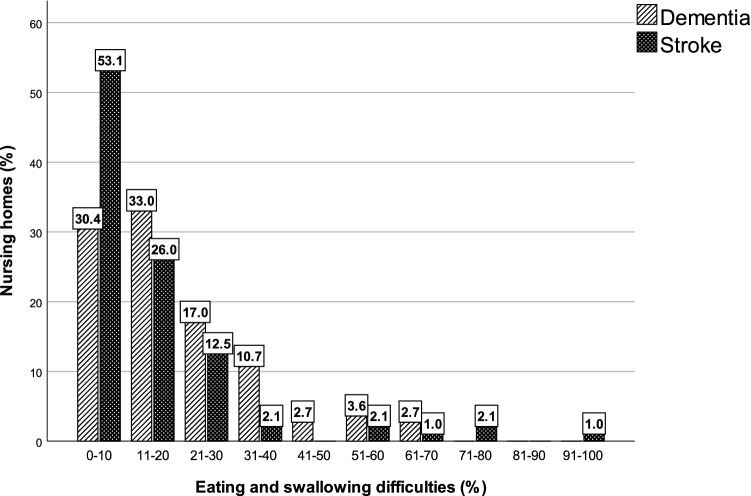
Estimated prevalence of eating and swallowing difficulties in people with dementia and stroke

The participants were asked to provide estimated figures on diagnostic groups with eating and swallowing difficulties. Figure 2 shows the relative numbers of nursing homes and the estimated range of difficulties for both dementia (*n* = 112 homes) and stroke (*n* = 96 homes). Prevalence estimates vary greatly, with 30.4% and 53.1% of nursing homes reporting an estimated prevalence of eating and swallowing difficulties as low as 0–10% in people with dementia and stroke patients, respectively.

**Fig. 3:**
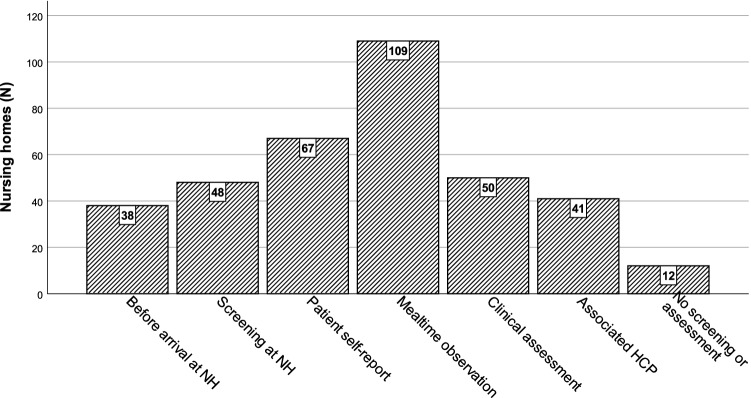
Types of screening and assessment for eating and swallowing difficulties in nursing homes

When asked about routines for screening and assessment of eating and swallowing difficulties (Fig. 3), 90.1% (109/121) of the respondents referred to mealtime observations as common practice, whereas another 55.4% (67/121) used residents’ self-reported complaints; 39.7% (48/121) of the nursing homes had implemented a screening, and 41.3% (50/121) performed further clinical assessment for dysphagia; 33.9% (41/121) referred to other health care professionals outside of the nursing home for reporting dysphagia; and 9.9% (12/121) did not screen or assess their residents for eating and swallowing difficulties at their nursing homes or refer to external health care professionals. Approximately half of the respondents (45.5%; 55/121) screened or assessed residents for eating and swallowing problems upon their first arrival at the nursing home. Whenever staff noticed a change in residents’ cognitive or physical functioning, screening and assessment was standard practice (73.6%; 89/121). Most homes did not have strict routines around planning screening and assessment for eating and swallowing difficulties, but 22.3% (27/121) of homes scheduled intermittent screening and assessment including a weekly routine (0.8%; 1/121), monthly routine (7.4%; 9/121), or yearly routine (14.0%; 17/121).

**Fig. 4:**
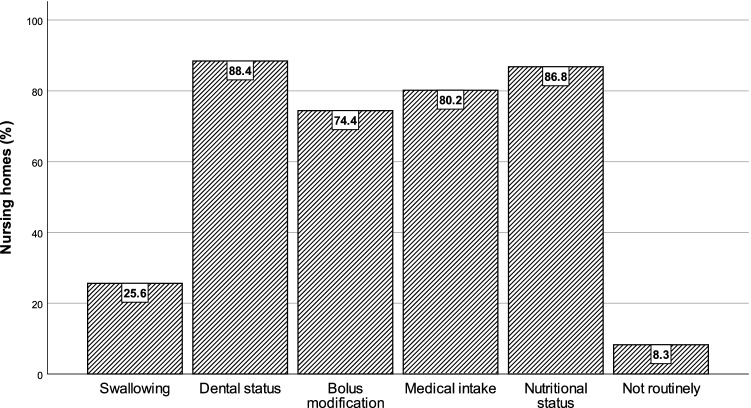
Routinely implemented screening procedures in nursing homes

In response to whether residents were being screened or assessed for swallowing function in particular (that is, not targeting, for example, oral intake, mealtimes or malnutrition), 25.6% (31/121) of all homes confirmed having these practices included in the daily routines at their nursing homes (Fig. 4). In addition, most nursing homes routinely screened for dental status (88.4%; 107/121) and nutritional status (86.8%; 105/121). The homes also screened for the need to adjust either medicine intake (80.2%; 97/121) or recommendations on oral intake and bolus modification (74.4%; 90/121). A total of 8.3% (10/121) of the nursing homes did not have any screening routinely implemented.

**Fig. 5:**
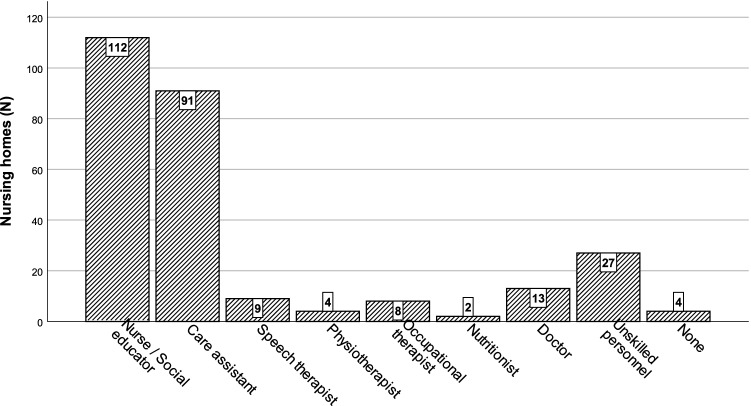
Staff responsible for screening and assessment for eating and swallowing difficulties

**Fig. 6:**
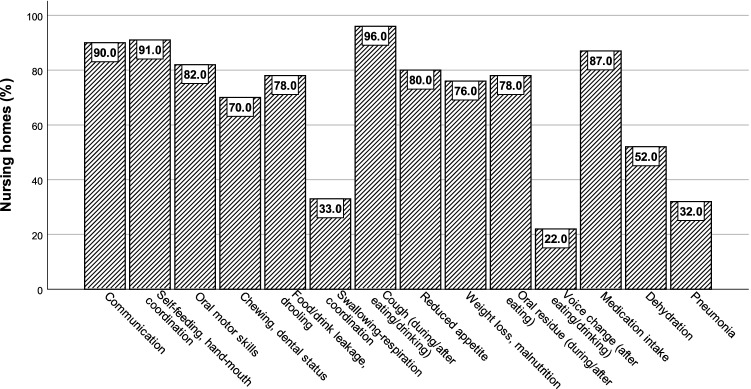
Observed challenges and difficulties in persons with eating and swallowing difficulties in nursing homes

Screening and assessment for eating and swallowing difficulties (Fig. 5) fell most frequently under the responsibility of nurses and social educators (92.6%; 112/121) or care assistants (75.2%; 91/121). In 22.3% (27/121) of the nursing homes, unskilled personnel were involved, and in 10.7% (13/121), doctors were involved. Other health care professionals, including speech therapists, occupational therapists, physiotherapists or nutritionists, were involved in 7.4% (9/121), 6.6% (8/121), 3.3% (4/121), and 1.7% (2/121) of the nursing homes, respectively. Figure 6 shows the observed variety in challenges and difficulties in people with eating and swallowing disorders as perceived by the respondents.

### Dysphagia Management and Clinical Practice in Nursing Homes

**Fig. 7:**
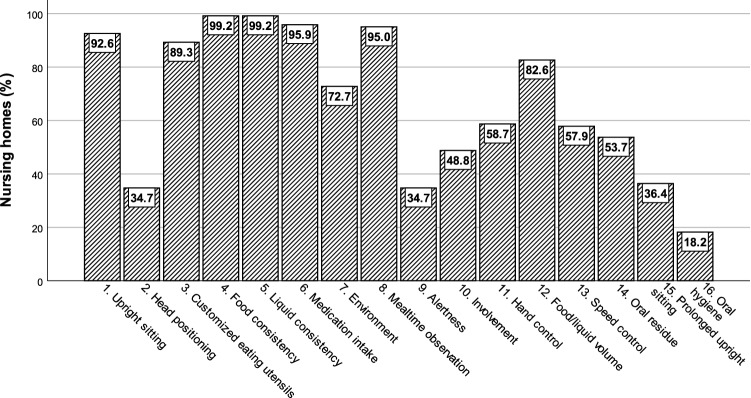
Strategies and routines for people with eating and swallowing difficulties in nursing homes

Nursing homes use many different strategies and routines to support people with eating and swallowing difficulties (Fig. 7). Almost all homes modified consistencies of liquids (99.2%; 120/121) and food (99.2%; 120/121), changed administration of medicine intake such as crushing tablets or replacing pills with liquid medicines (95.9%; 116/121), improved residents’ upright sitting posture (92.6%; 112/121), used customized mealtime utensils (89.3%; 108/121), and supervised mealtimes (95%; 115/121). Control of bolus size per bite or sip (82.6%; 100/121) and avoidance of distracting background activities or noise such as television or music (72.7%; 88/121) were also part of the homes’ clinical routines. Over 50% of all respondents confirmed that offering hand support during eating, checking for oral residue, controlling speed of oral intake, and having residents actively engaged in drinking and eating activities were all strategies incorporated into nursing home care. Less common practices across homes were adjusting head positioning (34.7%; 42/121), checking for residents to be well rested and alert during mealtimes (34.7%; 42/121), or allowing prolonged upright sitting after mealtimes for at least 15 min (36.4%; 44/121). Oral hygiene after meals was implemented in less than 18.2% (22/121) of all nursing homes. A total of 50.41.3% (61/119,121) of nursing homes had access to external experts (e.g., speech therapists or occupational therapists) for the treatment of eating and swallowing difficulties; 432.0% (520/119,121) of the homes relied on internal staff only, and 6.67% (8/119,121) had no knowledge about options for having external experts involved.

Residents with eating and swallowing difficulties usually had mealtimes under individual or group supervision of an assistant (95.0%; 115/121). Mealtimes were served either in a resident’s private room (70.2%; 85/121) or in the shared dining room (92.6%; 112/121). A minority of homes (5.0%; 6/121) did not have any special routines for the location or supervision of mealtimes for residents with difficulties. In general, meals for residents having eating and swallowing problems were prepared by the home’s own kitchen staff (73.6%; 89/121) and/or other nursing home personnel (75.2%; 91/121). A total of 22.3% (27/121) indicated that meals were prepared by external kitchen facilities. Homes used different guidelines or routines when implementing recommendations for eating, drinking or medication intake. The majority referred to dietary handbooks (57.9%; 70/121). Although some homes introduced food and liquid classification systems developed by their own staff (9.9%; 12/121), 28.1% (34/121%) did not have any system in place. One home reported using the International Dysphagia Diet Standardisation Initiative (IDDSI) [15].

### Dysphagia Training and Education

In general, staff at nursing homes were not required to undergo obligatory training or education in eating or swallowing difficulties (87.4%; 112/119; *n*_*MISSING*_ = 3). Three respondents did not know if education was obligatory, and 10.1% (12/119) confirmed obligatory staff training. The most common routine for upskilling staff was through clinical practice (onsite training) and supervision by a more experienced colleague (56.2%; 68/121). Staff also had access to theoretical training, including online courses (29.8%; 36/121), external expert workshops (11.6%; 14/121) or consultation by external health care professionals (13.2%; 16/121). At 19.8% (24/121) of the nursing homes, staff were not offered continuing professional development within this area. Similar results were found for specific training of kitchen staff in preparing modified diets for residents: mostly theoretical upskilling (32.2%; 39/121), onsite training (34.7%; 42/121) or external courses by experts (24.0%; 29/121). Only 9.9% (12/121) of homes had access to consultation by external professionals. In 13.2% (16/121) of homes, no training was offered, while 21.5% (26/121) of the respondents were unaware of educational initiatives for kitchen staff.

### Self-Perceived Quality of Dysphagia Care

**Fig. 8:**
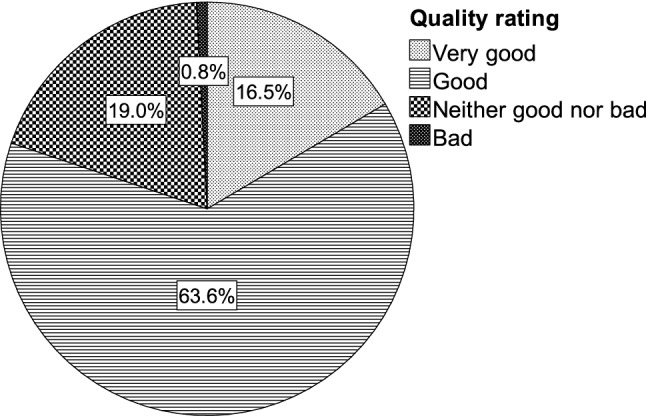
Self-perceived quality of care for people with eating and swallowing difficulties in nursing homes

At the end of the survey, the respondents were asked how they perceived the current quality of care for people with eating and swallowing difficulties in their nursing home (5-point scale ranging from very good to very bad). The majority of respondents (63.6%; 77/121) considered care in their nursing home to be of good quality (Fig. 8). Some twenty percent rated quality of care to be very good (16.5%; 20/121) or neither good nor bad (19.0%; 23/121). One respondent perceived quality of care as bad, whereas none of the respondents rated care as very bad.

### Group Differences

Group differences in dichotomized perceived quality of care ratings and frequency ratings on screening and assessment routines were investigated and stratified according to participant and nursing home characteristics (for example, participant professional background and location and size of nursing home). As univariate chi-square tests did not identify any variables for inclusion in multivariate regression analysis (all *p*-values ≥ 0.1), no regression analyses were performed. Instead, descriptive analyses were reported to describe survey responses.

## Discussion

The overall purpose of this study was to explore management and care pathways for elderly people with dysphagia in nursing homes across Norway. A national survey among all IPLOS-registered nursing homes in Norway was completed by 121 respondents, resulting in an overall response rate of 23.2%. The response rate between counties ranged from 6.9 to 36.1%. The Oslo region and Viken showed the lowest response rates (6.9% and 13.7%, respectively), possibly because many research initiatives may originate from Norway’s capital, targeting nearby counties such as Viken.

Overall, the results indicated substantial differences between homes in implemented routines and strategies for people with eating and swallowing problems. Differences were identified in screening and assessment, management and interventions, and training of nursing home staff. Despite these differences, the overall quality of care in nursing homes as perceived by the respondents was mostly rated between good and very good (80.2%; 97/121).

### Screening and Assessment

The prevalence of dysphagia in nursing home populations has been reported in the literature to vary between 13.4 [12] and 52.7% [2]. Although the majority of respondents were aware of eating and swallowing problems in residents, particularly in post-stroke people or people with dementia, the prevalence was estimated, in general, to be significantly lower than the data in the literature.

Clinical symptoms of OD are associated with an increased risk of malnutrition in nursing home residents [7]. One-year national registry data in Norway confirmed that 50.5% of all nursing homes screened residents for being at nutritional risk [13].

The most common routines for screening and assessing eating and swallowing problems were mealtime observations (90.2%) and resident self-reports (55.4%). Nevertheless, some 10% of the Norwegian nursing homes did not screen or assess residents for eating or swallowing problems or refer to external health care professionals. Furthermore, almost 10% did not have any routines implemented to screen for dental status and nutritional status or to adjust medicine intake, nutritional intake or bolus modification.

In approximately 25% of homes, unskilled personnel were involved in screening or assessing residents with eating and swallowing problems. In general, screening does not require high-skilled experts, but any staff involved should be trained in how to screen and how to interpret the results to improve the reliability of screening. Assessment, however, would assume trained experts’ involvement [16].

Most respondents were familiar with symptoms associated with eating and swallowing problems, although some challenges and difficulties were less known, such as voice changes after eating or drinking (18.2%), pneumonia (26.4%) or dehydration (43%). When asking specifically about swallowing, approximately 75% of all homes stated that residents’ swallowing function was not routinely screened for or assessed. This indicated that dysphagia screening is not common practice in most nursing homes. Additionally, even though our survey provided background information about the concept of dysphagia, respondents may have interpreted eating and swallowing problems as mainly a problem of malnutrition. This survey seemed to confirm the lack of awareness of dysphagia and tendency to underestimate the importance of identifying swallowing problems at an early stage by screening and assessment.

### Intervention

The nursing homes used a broad range of strategies and routines for people with eating and swallowing difficulties. With the exception of one single home, the other 120 homes used liquid and food modification as the most common strategy and routine. However, since approximately 75% of homes do not routinely screen or assess swallowing function or refer to external experts for screening or assessing for dysphagia, the implemented routine of bolus modification in nursing homes does not seem to be based on evidence-based clinical practice or adjusted to residents’ specific needs. Instead, the implementation of bolus modification may be standard practice for all residents and not restricted to people in need of modified food and liquids. In the literature, evidence can be found that increasing viscosity reduces the risk of airway invasion and that it is considered a valid management strategy for dysphagia [17]. However, more recent systematic reviews on whether thickening liquids and modifying foods are beneficial for adults with dysphagia could not support the use of texture-modified liquids or identify scientific evidence for modified foods [18]. Instead, the impact of bolus modification on health-related quality of life in patients with oropharyngeal dysphagia appears to be negative, with increased modification of food and fluids often correlating to a decreased quality of life [19]. As such, prescribing bolus modification in nursing homes may not be the best clinical practice for all residents.

Additionally, many homes did not use strategies or interventions such as adjusting head positioning (34.7%), checking for residents to be well rested and alert during mealtimes (34.7%), or allowing prolonged upright sitting after mealtimes for at least 15 min (36.4%). However, the lack of oral hygiene strategies after meals was more disconcerting. Only 18.2% of respondents confirmed having oral hygiene routinely in place after mealtimes. Oral hygiene care is associated with decreased odds of pneumonia in stroke patients [20, 21] and nursing home residents [22]. Research has also shown that a strict routine of oral care can reduce aspiration pneumonia in patients with oropharyngeal dysphagia [23]. In addition, systematic reviews have confirmed that people with dementia have worse oral health compared to older people without dementia with more frequent diseases of oral soft tissues and dental hard tissues [24, 25].

Furthermore, although most nursing homes did have special routines for location or supervision during mealtimes for residents with eating and swallowing problems, 5% of homes did not have any routines in place at all. This is concerning, especially since many residents may have cognitive problems in addition to dysphagia, thus increasing the risk of unsafe swallowing.

As in many other countries, the management of dysphagia in Norway falls within the area of expertise of speech therapists. As almost 50% of the homes did not have access to external experts such as speech therapists, the homes relied heavily on internal staff for supporting people with dysphagia. Only very few homes had speech therapists among their internal staff, demonstrating the need for training and upskilling internal staff to provide best evidence-based practice and care to residents with dysphagia.

### Education

In general, nursing home staff is not required to undergo obligatory training in dysphagia (87.4%). The most common routine for upskilling staff was through clinical practice and supervision by colleagues (56.2%), although almost 20% of homes did not offer any training in dysphagia. Similar results were obtained when asking about training in the preparation of modified diets for kitchen staff, even though approximately 75% of meals for persons with eating and swallowing problems were prepared by internal kitchen or other staff. The actual number of staff members not receiving any training (13.2%) may be much higher, as many respondents were unaware of educational initiatives for nursing kitchen staff (21.5%).

Almost 30% of homes did not have a classification system or common language to describe food and liquids. The combination of insufficient qualified personnel and lack of a classification system may result in unsafe mealtime experiences for people with dysphagia in nursing homes.

### Limitations

Although response-rate-induced bias may not seem to be much of a threat to the validity of questionnaires, it may have an impact on whether the responses represent people’s true states, attitudes, and behavior [26]. Small populations need relatively high response rates to establish confidence in generalizing results to the entire population [27]. Our study achieved a response rate of 23.2% based on 121 respondents out of 521 invited nursing homes, which is in line with many other surveys in health care (for example, [28, 29]). Although a higher response rate might have strengthened our results, we consider our respondents to be a representative selection of the targeted nursing homes. The current response rate may also reflect the lack of awareness of dysphagia in Norwegian nursing home settings.

Additionally, while aiming for adequate response rates, a balance was struck between survey depth and respondent burden. The current survey explored the management and care pathway for elderly people with dysphagia in Norwegian nursing homes. Future research needs to focus on additional details on, for example, frequencies of reported screening and assessment and intervention strategies.

Lastly, even though all respondents had knowledge about management and daily routines within their respective nursing home, participants’ educational and clinical backgrounds varied markedly, which may have influenced survey results. Future research should aim at ensuring greater representation from all main professional groups by including several participants from each nursing home.

## Conclusions

Substantial discrepancies in dysphagia management were identified between nursing homes across Norway. Residents were not routinely screened or assessed for swallowing problems in approximately 75% of all homes. Most nursing homes used a broad range of strategies and routines for people with eating and swallowing difficulties. Bolus modification, however, was standard practice, whereas oral hygiene strategies were lacking in over 80% of nursing homes. Nearly 50% of homes did not have access to external experts, including speech therapists. Although nursing home staff rated the overall quality of care for people with eating and swallowing problems as high, their rating seemed mainly based on care for malnutrition and not directly aimed at dysphagia. There is an obvious need for training and upskilling staff in Norwegian nursing homes and raising awareness of the serious consequences and comorbidities that can result from dysphagia.

## Supplementary Information

Below is the link to the electronic supplementary material.

Supplementary file1 (DOCX 25 KB)
