# Supplementary material for: Management of Dysphagia in Nursing Homes: A National Survey
Source: Dysphagia. 2021 Mar 4;37(2):266–76. doi: 10.1007/s00455-021-10275-7 (PMC8948132; doi:10.1007/s00455-021-10275-7)
Supplement: Supplementary file 1 — Supplementary file1 (DOCX 25 KB) [file 455_2021_10275_MOESM1_ESM.docx]

**Management of dysphagia in nursing homes: A national survey**

**Mina C.N. Engh^1,2^, Renée Speyer^1,3,4^**

^1^Department Special Needs Education, University of Oslo, Oslo, Norway

^2^Indre Østfold Municipality, Askim, Norway

^3^School of Occupational Therapy, Social Work and Speech Pathology, Faculty of Health Sciences, Curtin University, Perth, Australia

^4^Department of Otorhinolaryngology and Head and Neck Surgery, Leiden University Medical Centre, Leiden, The Netherlands

**NATIONAL SURVEY IN NURSING HOMES^a,b^**

***Participants***

1.1 What is your position at the nursing home where you work (e.g., clinical nurse, department manager, executive officer, director of care, head of allied health, health care professional)?

1.2 What is your educational / professional background (e.g., health management, health care profession, specialized health care profession)?

***Nursing home and staffing***

2.1 In which [Norwegian] county is your nursing home located?

2.2 What type of care do you provide at your nursing home (e.g., somatic care, geriatric or dementia care)?

2.3 How many places or beds are available in total?

2.4 What duration of care is provided at your nursing home (e.g., daycare, short-term stay, long-term stay)?

2.5 Which diagnostic groups of residents are eligible to use your services (e.g., residents with dementia, neurodegenerative patients, traumatic brain injury, stroke, head and neck or oesophageal cancer, or congenital neurological conditions)?

2.6 Which professionals are working in your nursing home (e.g., manager, nurse, social worker, nutritionist, speech therapist, physiotherapist, occupational therapist, care assistants, unskilled personnel)?

Of the professionals that you listed above, how many fulltime equivalent staff do you have?

***Screening and assessment for dysphagia***

3.1 How high would you estimate the percentage of residents (per diagnostic group) that have eating and swallowing difficulties?

3.2 How do you screen/assess residents for eating and swallowing difficulties (e.g., screening/assessment before arrival at nursing home, screening at arrival at nursing home, resident’s self-report or caregiver’s report, mealtime observation, clinical assessment, report by other health care professionals outside of the nursing home)?

3.3 How often do you screen/assess for eating and swallowing difficulties (e.g., at arrival, in case of change in resident’s cognitive or physical functioning, weekly routine, monthly routine, yearly routine)?

3.4 Who screens/assesses residents for eating and swallowing difficulties (e.g., nurse, social worker, nutritionist, speech therapist, physiotherapist, occupational therapist, care assistants, unskilled personnel)?

3.5 What challenges or additional difficulties do residents with eating and swallowing difficulties experience (e.g., problems in communication, self-feeding or eye-hand coordination, dental status, drooling, coordination between swallowing and respiration, coughing during eating or drinking, reduced appetite, weight loss or malnutrition, food residues in mouth after swallowing, changed or wet voice after drinking, problems with medicine intake, dehydration, pneumonia)?

3.6 What type of assessment is conducted routinely with residents at your nursing home (e.g., swallowing function, dental status, need to adjust the consistency of food and drink, need to adjust medicine intake [e.g., change of consistency, crushed tablets], nutritional status)?

***Dysphagia management and clinical practice in nursing homes***

4.1 Which strategies and routines do you use to support residents with eating and swallowing difficulties (e.g., improving residents’ upright sitting posture, adjusting head positioning, use of customized mealtime utensils, modification of food consistencies, modification of liquid consistencies [drink], change of medicine intake [e.g., change of consistence, crushed tablets], avoidance of distracting background activities or noise [e.g., television, music], mealtime observation [supervision], checking for residents to be well rested and alert during mealtimes, offering hand support during eating, control of bolus size per bit or sip, checking for food residues in mouth, controlling speed of oral intake, having residents actively engaged in drinking and eating activities, allowing prolonged upright sitting after mealtimes for at least 15 minutes, oral care after meals)?

4.2 Do you have access to external clinical professionals for assessment and treatment of eating and swallowing difficulties?

4.3 Where do most of your residents with eating and swallowing difficulties have their meals (e.g., resident’s private room with an assistant, shared dining room together with other residents and supervision, shared dining room together with other residents and an assistant, shared dining room separated from other residents, shared dining room together with other residents with eating and swallowing difficulties, shared dining room together with other residents [no special arrangements])?

4.4 Who is responsible for preparing meals for residents with eating and swallowing difficulties? (e.g., external kitchen facilities, nursing home kitchen staff, residents’ relatives)?

4.5 Which classification system do you use for residents who need adapted consistency of food, drink and or medicine (e.g., dietary handbooks, International dysphagia diet standardisation initiative [IDDSI], classification system developed by nursing home staff)?

***Dysphagia training and education***

5.1 Do you have obligatory educational training for staff in eating and swallowing difficulties at your nursing home?

5.2 Which opportunities for professional development in eating and swallowing difficulties are available to staff (e.g., theoretical upskilling [e.g., webinars, internal courses], onsite training [e.g., workshop or supervision by experienced staff], external expert course on eating and swallowing difficulties)?

5.3 Do you upskill kitchen staff at your nursing home in preparing meals for residents with eating and swallowing difficulties (e.g., theoretical upskilling (e.g., webinars, internal courses), onsite training [e.g., workshop or supervision by experienced staff], external expert course on eating and swallowing difficulties)?

***Self-perceived quality of dysphagia care***

6.1 How would you rate the quality of care for people with eating and swallowing difficulties at your nursing home?

^a^ Original survey developed in Norwegian

^b^ Survey headings refer to corresponding paragraphs in the discussion section of the article.

*Note*. The original questionnaire consisted of multiple choice questions (17 items), matrix questions (4 items), a numeric textbox question (1 item) and one ordinal scale (1 item). The survey contained short explanations where appropriate on topics such as ‘dysphagia’ or ‘screening and assessment’. As participants were expected to have different educational backgrounds and possibly be less familiar with medical terms, the use of professional jargon was avoided. Participants could elaborate on questions using open comment boxes throughout the survey.
